# Poloxamer-based drug delivery systems: Frontiers for treatment of solid tumors

**DOI:** 10.1016/j.mtbio.2025.101727

**Published:** 2025-04-02

**Authors:** Mehdi Pourbakhsh, Masoud Jabraili, Morteza Akbari, Mehdi Jaymand, Rana Jahanban Esfahlan

**Affiliations:** a Department of Medical Biotechnology, Faculty of Advanced Medical Sciences, Tabriz University of Medical Sciences, Tabriz, Iran; b Department of Molecular Medicine, Faculty of Advanced Medical Sciences, Tabriz University of Medical Sciences, Tabriz, Iran; c Nano Drug Delivery Research Center, Health Technology Institute, Kermanshah University of Medical Sciences, Kermanshah, Iran; d Student Research Committee, Kermanshah University of Medical Sciences, Kermanshah, Iran

**Keywords:** Pluronics, Poloxamers, Cancer therapy, Drug delivery

## Abstract

Pluronics or poloxamers are a type of triblock copolymer. These non-ionic molecules consist of a hydrophobic block embedded in two hydrophilic parts. Pluronics have become favorite materials for use in the field of biomedical research due to having favorable physicochemical and biological properties such as amphiphilicity, solubility in ionic and non-ionic solutions, biocompatibility, biodegradability, self-assembly and low toxicity. The scope of these applications can vary from tissue engineering to drug delivery. One of the important uses of pluronics is to deliver drugs to various cancer cells. Herein we first provide an overview on variety of ploronic biomaterials. And then intensively evaluate their potential as drug delivery systems (DDSs) for treatment of solid tumors with special focus on breast cancers. After explaining the pros and cons of pluronics, the current status in clinical settings and future prospects are highlighted.

## Graphical abstract

**Figure:**
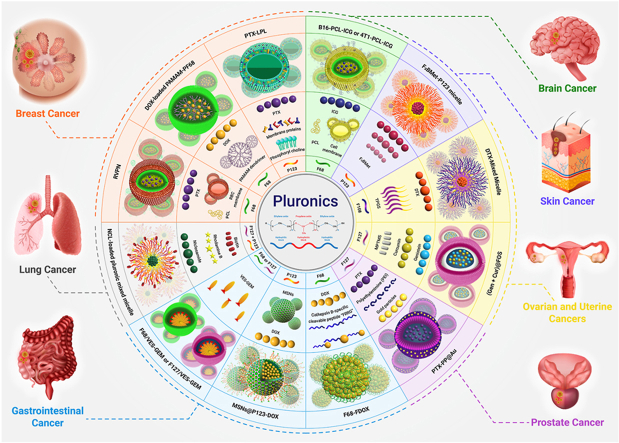

## 1 Introduction

Pluronics, also known as poloxamers, are synthetic and nonionic triblock copolymers. These copolymers are amphiphilic in nature and consist of poly(propylene oxide) (PPO) and poly(ethylene oxide)(PEO) polymer blocks. pluronics have a three-block structure in which a hydrophobic unit (PPO) is placed between two hydrophilic units (PEO) in the form of (PEO)_n_-(PPO)_m_-(PEO)_n_. Poloxamers are soluble in polar and non-polar solvents [[1], [2], [3], [4]]. The biocompatibility and physicochemical properties of pluronics have made them a promising tool in biomedical applications, especially in the field of drug delivery. According to the studies that have been conducted, no acute or chronic toxicity caused by these polymers has been reported. Therefore, these molecules can be considered as biocompatible and safe agents for administration. Some different pluronic polymers have received Food and Drug Administration (FDA) approval for pharmaceutical use and even intravenous injection [5,6]. Pluronics in aqueous media showed the micellization through self-assembly at concentrations above critical micelle concentration (CMC). Self-assembled pluronic micelles in aqueous solutions formed a core-shell structure having a diameter range from 10 nm to 100 nm [7,8]. The core-shell structure consists of a hydrophobic core and a hydrophilic shell [9]. The existence of the core and shell structure makes it possible to formulate hydrophobic drugs inside them [10]. Another important feature that has caused increasing interest in pluronics as a nanocarrier in biomedical studies is the flexibility and tunability of their chemical and physical properties [[11], [12], [13]]. Also, the surface of pluronic nanocarriers can be functionalized through physical and chemical connection with other molecules and they can be specific for different molecular purposes in the target cells [14]. In addition to these features and acting as a carrier, pluronics have various biological effects in the body, especially against cancer cells [12,15]. Among these biological activities are the ability of these polymers to incorporate into membranes, then move into the cell and influence various cellular functions such as mitochondrial respiration, ATP production, apoptosis signal transmission, efflux pump activity and gene expression [[16], [17], [18]]. These biological activities enable pluronics to act as therapeutic agents and make cancer cells (especially multi-drug resistant (MDR) tumors) more sensitive to various anticancer agents [15,[19], [20], [21], [22], [23]]. Due to these characteristics, the use of pluronics in cancer studies has attracted a lot of attention in recent years.

In this review article, first, the physicochemical and biological properties of pluronic copolymers that make them suitable for use in drug delivery will be discussed. Then, the studies in which different formulations based on pluronics are used as carriers for anticancer agents in different cancers will be mentioned.

## 2 Physicochemical properties of pluronics and drug loading capacity

**Fig. 1:**
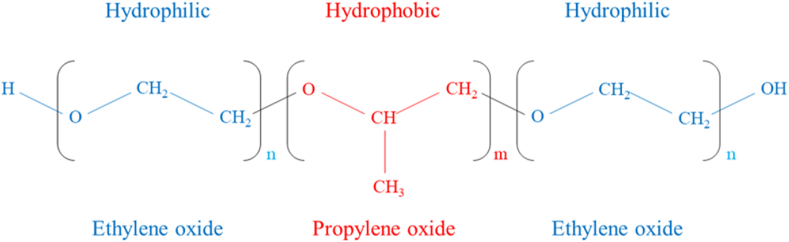
Triblock structure of pluronic copolymers.

Pluronic polymers have amphiphilic properties and are in the form of white, waxy, tasteless and odorless granules [4,11,18,24]. Pluronics have a triblock structure consisting of PEO and PPO blocks. In this structure, PEO and PPO are arranged in the form of (PEO)_n_-(PPO)_m_-(PEO)_n_ [3,19,25] (Fig. 1). The PEO blocks that are placed on both sides of the copolymer structure have created hydrophilic parts, and the PPO block that is placed between the two hydrophilic parts is responsible for the hydrophobic (or lipophilic) property of the molecule [26]. Depending on the number of *m* and *n* and depending on the percentages of PEO and PPO in the structure of the polymer, the type of pluronic and its physical properties will be different. So far, several types of pluronic copolymers with different molecular weights (varies from 1000 to 20000 g/mol) have been synthesized and are commercially available [[27], [28], [29]]. Some of them and their important characteristics are listed in Table 1. The difference in the number of EO and PO units in different pluronics also causes a distinct and different hydrophilic-lipophilic balance (HLB) value among them [4].

**Table 1:** Some types of pluronic and their physicochemical properties^a^.

Pluronic type	Molecular weight (Da)	Average no. of EO units (n)	Average no. of PO units (m)	CMC (M)	HLB
F68	8400	152.73	28.97	4.8 × 10 ^−4^	29
F87	7700	122.50	39.83	9.1 × 10 ^−5^	24
F88	11400	207.27	39.31	2.5 × 10 ^−4^	28
F108	14600	265.45	50.34	2.2 × 10 ^−5^	27
F127	12600	200.45	65.17	2.8 × 10 ^−6^	22
L35	1900	21.59	16.38	5.3 × 10 ^−3^	19
L61	2000	4.55	31.03	1.1 × 10 ^−4^	3
L64	2900	26.36	30.00	4.8 × 10 ^−4^	15
L81	2750	6.25	42.67	2.3 × 10 ^−3^	2
L121	4400	10.00	68.28	1.0 × 10 ^−6^	1
P85	4600	52.27	39.66	6.5 × 10 ^−5^	16
P103	4950	33.75	59.74	6.1 × 10 ^−6^	9
P105	6500	73.86	56.03	6.2 × 10 ^−6^	15
P123	5750	39.20	69.40	4.4 × 10 ^−6^	8

a Data obtained from references [13,20,27,[29], [30], [31], [32]].

As mentioned earlier, one of the important features of pluronic polymers is their tendency to self-aggregate and form different structures such as micelles, in water solutions. The formation of these structures is affected by various factors, both in the structure of the pluronic molecule itself and environmental factors. Among the influential factors in the micellization of pluronic units, we can mention the parameters of critical micellar temperature (CMT) and CMC. These parameters themselves are affected by factors such as pluronic molecule composition, PPO content and molecular weight. CMT increases with increasing hydrophilicity of pluronic polymer. If the PPO/POE ratio is constant, CMC and CMT will decrease as the molecular weight of the copolymer increases. Also, depending on the composition, temperature and concentration, pluronics can form other aggregate structures such as gels, lamellar phases and rod-like structures with hexagonal symmetry, in addition to micelles [3,11,[33], [34], [35], [36], [37]].

The self-assembly capability of pluronics in different conditions and especially the formation of micelles by them makes these biocompatible polymers very suitable for various biomedical applications such as drug delivery, gene delivery, tissue engineering, etc [10,11,[38], [39], [40], [41], [42]]. These aggregated structures create a hydrophobic environment inside and a hydrophilic shell around (core-shell aggregates). Therefore, it is possible to load hydrophobic drugs in a lipophilic environment inside these structures (especially micelles). Also, the hydrophilic shell protects the drug loaded in the core from degradation by the environment and causes the cargo to be transported in the hydrophilic environment [10,38,43,44]. Additionally, due to pluronic's thermoresponsive properties, it can also be used to develop *in situ* forming hydrogels [45]. *In situ* forming hydrogels are suitable vehicles for the delivery of drugs for both local treatment and systemic effects [5,[46], [47], [48]].

It is possible to modify and optimize the physicochemical properties of pluronics according to the desired therapeutic goals. This feature makes it possible to develop pluronic-based targeted delivery systems by adding targeting moieties and other molecules on the surface of pluronic nanoparticles (NPs). Also, pluronic copolymers can be easily combined and conjugated with some other widely used polymers. This has led to the creation of mixed delivery systems containing pluronic and other polymeric components (such as poly(lactic-co-glycolic) acid (PLGA), Polylactic acid (PLA), chitosan, etc.) that are very common [6,14,49,50].

In addition, pluronic-based systems can be used either for single drug delivery or for combined drug regimens. These combined delivery systems provide a platform for pharmacodynamic synergy and drug efficacy enhancement [12,51,52]. Another parameter that is very important in the drug delivery application of pluronic micelles is their drug loading capacity. The drug loading capacity of these polymers can be different depending on the structure of the drug itself, as well as the pluronic structure and its hydrophobic block properties. pluronic polymer concentration, solution temperature, molecular weight, and the size and ratio of hydrophilic and hydrophobic blocks are among other factors that affect the drug loading capacity [3,10,11,[53], [54], [55]]. Although hydrophobic pluronics have greater drug dissolution capacity than the hydrophilic types of these polymers through the formation of layered structures, but these structures have larger size and are less stable. To overcome this problem, researchers in various studies have formulated micelles that are created from a mixture of hydrophobic and hydrophilic pluronics. These binary micelles are often small in size and highly stable. They also have a higher loading capacity than hydrophilic copolymers [[56], [57], [58]]. The hydrophobicity/lipophilicity of drugs, which depends on the structure of the drug itself and is defined by the *partition coefficient* (*P*), is another important factor in the dissolution of hydrophobic drugs [59]. It has also been found that as temperature and ionic strength increase, drugs are better dissolved by pluronics with low HLB [60].

Kinetics of drug release is another important parameter in the application of Pluronic copolymers as drug carriers. Drug release from polymeric micelles depends on both the drug diffusion rate from intact micelles and the stability and biodegradability of the micelles [61,62]. Pluronic is not biodegradable and its release kinetics mainly depends on the interaction between the drug and the pluronic micelle core, the structure and concentration of the drug and its position inside the micelle. Although the strong interactions between the core and the drug increase the loading capacity, it slows down the drug release rate. Therefore, due to the fact that hydrophobic drugs are placed in the micelle core and interact strongly with it, the release is slow for them. But in connection with hydrophilic drugs, most of which are located at the interface of core and shell and do not have strong interactions with the hydrophobic core, explosive drug release often occurs [11].

Studies conducted to investigate the effect of solution pH on the accumulation of pluronic copolymers have shown that an increase in pH can lead to an increase in CMT in some types of pluronics. Also, very high acidity causes the destruction of these copolymers at higher temperatures [60,63]. To increase the pH-sensitivity of pluronic-based carriers, they can be conjugated with molecules that are sensitive to pH [64].

## 3 Biological properties of pluronic

Pluronics are biocompatible and have low immunogenicity. They are protected from phagocytosis by their PEO blocks and can remain in the bloodstream until they reach the target tissue. Also, their lower molecular mass compared to other polymers causes better filtration and removal of these molecules through the excretory system. Based on pharmacokinetic data, their clearance through the kidney has been revealed. Today, these polymers are considered as inactive molecules with low cytotoxicity. So that a significant number of these polymers (such as F127, F68, etc.) are classified by the FDA as "generally recognized as safe". Also, due to these features, some pluronics such as F127 have been approved by the FDA for various pharmaceutical applications [[65], [66], [67]].

It is known that pluronic copolymers, in addition to being drug carriers, have various therapeutic effects, especially on cancer cells. In the field of non-cancer applications, for example, it has been determined that pluronic F127 can be used to increase the rate of wound debridement of dry necrotic tissue, burn healing, other tissue engineering applications, and also as an immunoadjuvant (in the form of formulation with some emulsions) [[68], [69], [70], [71], [72], [73]]. Some formulations of this polymer can prevent adhesion after surgery or reduce the area of adhesion [[74], [75], [76]]. PF127 also accelerates the sealing of the permeabilized cell membrane during electroporation and thus prevents cell necrosis [77]. In the field of cancer treatment, pluronic copolymers, in addition to changing the pharmacokinetic properties of chemotherapeutics and increasing their bioavailability, can improve the response of tumor cells to these drugs and increase the efficiency of anticancer agents [10,11]. Some pluronic molecules, *per se*, can make MDR cells more sensitive to chemical drugs [18,[77], [78], [79], [80]]. These effects are mainly caused by inhibition of drug efflux transporters such as multidrug resistance proteins and P-glycoproteins [16,19]. Pluronics can alter mitochondrial respiration, apoptosis signal transduction and gene expression, also reduce ATP production in cancer cells [16,17]. Pluronic-based micelles have also been shown to have the ability to cross the blood brain barrier (BBB) [81].

## 4 Advanced pluronic-based drug delivery systems for treatment of solid tumors

Pluronics potentially have desirable properties that can make them an interesting candidate for use in cancer research. For this reason, there are a large and increasing number of studies related to the use of pluronics in the field of cancer research and cancer treatment. In this section, we will describe and review a number of these studies that are related to breast, lung, gastrointestinal system, prostate, ovary and uterus, and skin cancers, separately.

### 4.1 Breast cancer

**Fig. 2:**
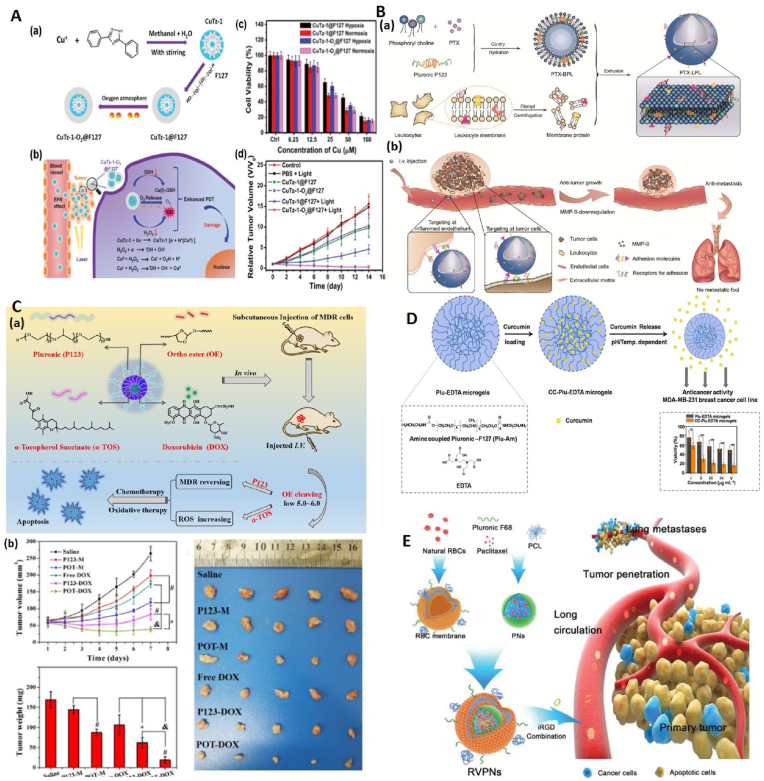
**Different ploronic based nanocariers for breast cancer therapy. A** (a). Synthesis of CuTz-1-O2@F127 nanoparticles and (b). Release of Cu(I) and O_2_ from CuTz-1-O2@F127 nanoparticles inside tumor cells and its effect on increasing PDT efficiency. (c). The PDT effect on 4T1 cells under 808 nm laser irradiation in vitro (d). Relative tumor volume of mice. Adapted from Ref. [84], Copyright (2019), John Wiley and Sons. **B,** (a). Formulation steps of LPL nanovesicle loaded with PTX and (b) its anti-metastasis efficacy in in vivo. Adapted from Ref. [85] with permission, Copyright (2019), Royal Society of Chemistry. **C,** (a). The structure and constituents of pH-sensitive POT-DOX micelles and their anticancer effects on MDR cancer cells through chemotherapy and oxidative therapy. (b) In vivo efficacy in mice. Adapted from Ref. [89] with permission, Copyright (2020), Elsevier. **D,** Plu-EDTA microgels structure, curcumin loading process and its release from these microgels. The graph shows the effect of Plu-EDTA microgels and curcumin-loaded Plu-EDTA microgels (CC-Plu-EDTA microgels) on the viability of MDA-MB-231 breast cancer cells. Adapted from Ref. [95] with permission, Copyright (2021), Elsevier. **E,** The structure and components of RVPNs. RVPNS, in combination with iRGD, increases the period of blood circulation, penetration into the tumor depth, and lung metastasis inhibition. (PCL: Polycaprolactone, PNs: hybrid polymeric NPs). Adapted from Ref. [96] with permission, Copyright (2016), John Wiley and Son.

Breast cancer is still one of the most common cancers in the world, and despite the advances made in conventional treatments, it still has a high mortality rate [82]. In this section, a number of studies will be discussed regarding the use of pluronics in the treatment of breast cancers (Fig. 2).

In 2017, Gold nanoparticle-coated pluronic-b-poly(L-lysine) nanoparticles (pluronic-PLL@Au NPs) were synthesized by Ying Sun and colleagues to deliver chemotherapy drug paclitaxel (PTX) to MDA-MB-231 breast tumor cell line for chemo-photothermal therapy. The results of calcein-AM and MTT assays showed that pluronic-PLL@Au NPs loaded with PTX have higher cytotoxicity on the mentioned cell line compared to the free drug. Also, during in vitro and in vivo studies, an obvious temperature response was recorded for pluronic-PLL@Au NPs, and it was found that the combination of chemotherapy and photothermal therapy (PTT) can be more effective than either of them alone. Also, blood compatibility and in vitro cytotoxicity assays confirmed the high biocompatibility of pluronic-PLL@Au NPs [83].

One of the new treatment methods in cancer treatment is photodynamic therapy (PDT). The presence of insufficient oxygen (O_2_) in the tumor microenvironment as well as the overexpression of glutathione (GSH) are factors that limit the effectiveness of this method. In the study of Xuechao Cai et al., a therapeutic platform based on pluronic CuTz-1-O_2_@F127 was used as a strategy to overcome these limitations (Fig. 2A). This platform has a metal-organic framework (MOF) and under Near-infrared (NIR) irradiation and in the presence of H_2_O_2_, this Cu(I)-based MOF is capable of a *Fenton-like reaction* and produces •OH and O_2_. Also, the release of O_2_ loaded in CuTz-1-O_2_@F127 nanoparticles results in further reduction of intracellular hypoxia. In addition, Cu(I) in CuTz-1@F127 can reduce excess GSH by reacting with intracellular GSH. The results of this study also showed the high antitumor efficacy of these nanoparticles through synergistic effect in 4T1 tumor-bearing mice under 808 nm laser irradiation. Biodegradability of nanoparticles was also investigated and confirmed. In vivo biodistribution and excretion experiments demonstrated the proper excretion of these nanoparticles through feces and urine [84].

Qinyue Chen et al. used pluronic polymer to synthesize leukocyte-mimicking pluronic-lipid nanovesicle hybrids (LPL) and investigated its inhibitory effects on the growth and metastasis of breast cancer (Fig. 2B). LPLs were created through the integration of membrane proteins extracted from leukocytes into membrane-like vesicles resulting from P123 hybridization in the lipid bilayer. Paclitaxel was loaded inside these vesicles as a model drug. Considering the bio-targeting ability of leukocytes and the reducing effect of P123 on matrix metalloproteinases (MMPs), the results of in vitro assays revealed increased cell uptake and anti-metastasis effect by these vesicles. Biodistribution assays also showed their high tumor targeting ability. In addition, during in vivo studies, it was observed that PTX-loaded LPL (PTX-LPL) has a high inhibitory effect on tumor growth and reduces the metastatic rate of tumor foci in lung tissue. In addition, PTX- LPL decreased the amount of MMP-9 and neutrophils in the tumor as well as in the lung [85].

Pluronic copolymers have been investigated as nanocarriers in inhibiting angiogenesis and metastasis of triple negative breast cancer (TNBC). TNBC is a very aggressive and metastatic type of breast cancer that is resistant to most existing treatments. Saurav Bhattacharya et al., used nanoparticles obtained from a mixture of hyaluronic acid (HA)-conjugated P123 and F127 pluronics to deliver thymoquinone (TQ) to TNBC cells; these nanoparticles called as HA-TQ-NPs. The average size of nanoparticles was 19 nm and their stability period at room temperature was 4 months. It was observed that applied HA-TQ-NPs nanoparticles have toxic effects on TNBC cells, while they do not cause any toxicity to normal cells. It was also found that HA-TQ-NPs reduce the ability to migrate and metastasize in this type of breast cancer. Further studies showed that these effects occur through up-regulation of microRNA-361. In addition, the pro-apoptotic and anti-angiogenic effects of these nanoparticles were also observed. In vivo studies on both MDA-MB-231 xenograft chick embryos and 4T1 mammary solid tumor model also confirmed the mentioned results [86].

Yi Zhao et al. used the micelles obtained from the combination of L61 and F127 pluronics, which were called SP polymers, to make cancer stem cells (CSCs) more sensitive to doxorubicin (DOX) in triple negative breast cancer. In the mentioned study, CSCs were derived from MDA-MB-231 and MDA-MB-468 cell lines and were characterized by features such as high epithelial specific antigen (ESA), high expression of CD44, and low levels of CD24. These cells were resistant to free DOX and showed increased invasiveness and tumorigenicity compared to the parent cancer cells and their non-CSC counterparts. However, it was found that polymeric micelle-based DOX (SP1049C) could reduce the expression of breast cancer resistance protein (BCRP/ABCG2) in CSCs and inhibit its functional activity in these cells. Also, the results of in vitro studies showed that compared to the free drug, SP1049C had higher cytotoxicity and potency in reducing the colony formation of CSCs. Further, the study on animal models containing tumors derived from CSCs confirmed these results. Therefore, it was concluded that SP1049C is an active compound against CSCs and has potential in the treatment of TNBC [87].

Multidrug resistance is one of the serious challenges in the clinical treatment of cancer, which prevents the proper therapeutic efficacy of anticancer drugs. To overcome MDR and increase the efficacy of chemotherapy, Xu Cheng et al. developed new polymeric prodrug micelles using F127 and P123 pluronic copolymers. These hybrid micelles named FBP-CAD were composed of phenylboric acid (PBA)-modified F127 and doxorubicin-grafted P123 (prodrug group). The micelles had two important characteristics: tumor targeting (due to the presence of PBA, which acts as an active targeting group) and acid sensitivity (due to the β-carboxylic amides bonds). FBP-CAD was stable in neutral environment, but drug release was accelerated in mild acidic conditions due to cleavage of β-carboxylic amides bonds. The results obtained from the in vitro studies showed that FBP-CAD increases cellular uptake and drug concentration in MCF-7/ADR cells through the targeting ability of PBA and the anti-MDR effect of P123 pluronic. Also, other assays revealed that FBP-CAD has the highest cytotoxicity on these cells compared to free DOX and other formulations (P123-CAD and FP-CAD). In addition, the study on MCF-7/ADR bearing-mice determined that these micelles facilitate drug accumulation in tumor sites and reduce drug-induced side effects in normal organs. The synergistic effect of active targeting and reversal of MDR leads to the highest inhibition of tumor growth in these mice [88].

To overcome multidrug resistance in breast cancer, Cheng et al. designed pH-sensitive pluronic micelles with synergistic effects of oxidative therapy. To synthesize these nanoparticles, pluronic P123 was modified with α-tocopherol succinate (α-TOS) through an acid-sensitive ortho ester (OE) linkage to form a pH-sensitive copolymer (POT). Then the micelles were synthesized through the self-assembly process and DOX was loaded inside these micelles as an anticancer drug. POT micelles had a high drug loading efficiency (82.59 ± 2.34 %) and the size of these nanoparticles was 80 nm. The results of in vitro experiments on MCF-7 and MCF-7/ADR breast cancer cell lines indicated that POT micelles can significantly reverse MDR by inhibiting efflux pumps (mediated by P123). These nanoparticles can also cause the production of reactive oxygen species (ROS) mediated by α-TOS, which leads to increased cytotoxicity and apoptosis in MDR cells. Also, the results of in vivo studies determined that DOX-loaded POT micelles (POT-DOX), compared to free DOX and P123-DOX, have the highest drug accumulation at the tumor site and the strongest inhibition of tumor growth. Pathological investigations showed that POT-DOX can induce apoptosis or necrosis at the tumor site without causing obvious damage to normal tissues (Fig. 2C) [89].

In another study, pluronic F68 (PF68)-attached polyamidoamine (PAMAM) dendrimer conjugates were used as DOX carriers to overcome drug resistance in breast cancer. In this study, a series of PAMAM-PF68 conjugates were designed and DOX was loaded inside them. Then, their antitumor activity against MCF-7/ADR cells, spheroids and tumors derived from these cells was investigated using different tests. The results showed that the conjugates loaded with DOX increase the antitumor activity in vitro and in vivo compared to the free drug. After escaping from the endosome/lysosome, the drug enters the cell nucleus and by affecting the regulation of gene expression in the nucleus as well as the regulation of mitochondrial function in the cytoplasm, it causes a significant increase in apoptosis [90].

In a 2019 study, Nguyen et al. used folate-conjugated chitosan-pluronic P123 nanogels (CP-FA) to co-delivery of hydrophobic drugs PTX and curcumin (Cur) to MCF-7 breast cancer cells. These drugs were loaded into the nanogels through a self-assembly process by hydrophobic interactions between the drugs and hydrophobic segment of the pluronic P123. The size of the obtained nanoparticles was 16.27 ± 2.01 nm and drug loading efficiency was reported 97.82 ± 0.48 for Cur and 98.63 ± 0.42 for PTX. Also, CP-FA nanogels showed controlled release of drugs for a long period of time. Then, the anticancer activity of CP-FA, free PTX, PTX-CP-FA, PTX/Cur-CP-FA and Cur-CP-FA compounds on MCF-7 cells was investigated using a sulforhodamine B (SRB) colorimetric assay. Based on the obtained results, it was found that PTX/Cur-CP-FA has a higher anticancer effect than PTX-CP-FA and Cur-CP-FA compounds, which indicates the synergistic effect of PTX and curcumin drugs. However, this study was among the few studies in which the free drug is more cytotoxic than a drug-loaded platform. It was found that free PTX has better cytotoxicity than other mentioned compounds. This was due to the delivery of PTX into the cytosol through passive diffusion (compared to the endocytic pathway for drug-loaded platforms), which could cause a rapid drug effect. Another reason was the slow release of PTX (19.46 % and 13.05 % at pH 5.6 and pH 7.4 after 48 h, respectively) from the nanoparticles [91].

Lin et al. synthesized pluronic-chitosan-folate nanomicelles containing quantum dots (QD) and DOX and investigated their toxicity on MCF-7 cancer cells. In addition, due to the presence of quantum dots in these nanoparticles, their potential in bioimaging was also studied. In this study, two types of QDs were used for comparison: zinc oxide (ZnO) and cadmium telluride (CdTe). In order to synthesize the mentioned compounds, pluronic micelles containing QDs and DOX were first prepared. Then the surface of these nanomicelles was modified with folate-conjugated chitosan to obtain QD-pluronic-chitosan-folate nanoparticles containing DOX. Cytotoxicity test results showed that both DOX-loaded ZnO-pluronic-chitosan-folate (ZnO-Plu-Fc-Chi) and DOX-loaded CdTe-pluronic-chitosan-folate have higher cytotoxicity against MCF-7 cells than free DOX. It was also found that CdTe quantum dots embedded inside nanoparticles can effectively show fluorescence during in vivo studies. Therefore, these nanoparticles have the potential to be simultaneously used for drug delivery and targeted cancer therapy as well as bioimaging [92].

Vorinostat is a histone deacetylase inhibitor that is used in the treatment of certain cancers and has been approved by the FDA. However, its relatively hydrophobic structure and consequently its limited aqueous solubility, low permeability, formation of aggregates in blood vessels and suboptimal pharmacokinetics make its delivery to cells difficult. To overcome these limitations, Mohamed et al. used pluronic micelles to deliver vorinostat to cancer cells. In this study, vorinostat was loaded separately in pluronic F127 (PF127) and pluronic F68 (PF68) micelles and these formulations were optimized based on the drug entrapment efficiency. The drug loading efficiency was reported as 99.09 ± 2.16 % and 94.19 ± 2.37 % for PF127 and PF68 micelles, respectively, when the ratio of drug to polymer was 1:50. The average diameter of nanoparticles was 72.61 ± 10.66 nm for PF68 and 91.88 ± 10.70 nm for PF127. It is also found that compared to PF68 micelles, drug release from PF127 micelles is slower and they show long-term release at pH 7.4. The cytotoxicity of drug-containing micelles on HepG2, Caco-2 and MCF-7 cancer cell lines was investigated in vitro and compared with the cytotoxicity of free drug. The obtained results showed that vorinostat-loaded PF127 micelles have the highest cytotoxicity on cancer cells compared to other compounds, and this enhancement of cytotoxicity was more pronounced on MCF-7 cells. Also, in vivo studies on mice bearing *Ehrlich Ascites Carcinoma* revealed that drug-containing PF127 micelles, compared to PF68 micelles and free drug, have a higher ability to inhibit tumor growth and significantly reduce liver and kidney toxicity [93].

Fisetin (FS) is a natural flavonoid that has been shown to have anticancer properties. To overcome the limitation of weak aqueous solubility of FS, Pawar et al. developed FS-loaded pluronic F127 (PF127)-folic acid (FA) conjugated micelles (FS-PF127-FA). In the first step, FA was conjugated to PF127 by carbodiimide crosslinker chemistry, and then FS-PF127-FA micelles were synthesized by thin-film hydration method. The drug loading efficiency was reported as 82.50 ± 1.78 % and the average diameter of nanoparticles was 103.2 ± 6.1 nm. In vivo studies on rats showed that FS-PF127-FA micelles increased the bioavailability of FS by 6-fold compared to free FS through increased circulation time and slower elimination from plasma. Also, no specific tissue toxicity caused by these micelles was observed in rats. In addition, in vitro studies revealed that FS-PF127-FA micelles show more cytotoxicity on MCF-7 breast cancer cell line than FS-PF127 and free FS. Therefore, FS-PF127-FA micelles can improve FS therapeutic efficiency by increasing solubility, increasing bioavailability and active targeting of MCF-7 cells by FA [94].

In the study conducted by Kulkarni et al., temperature sensitive pluronic F-127 based microgels were used to optimize the transfer of curcumin to breast cancer cells. In order to synthesize these microgels, first the pluronics were modified with amine groups and then they were connected to ethylenediaminetetraacetic acid (EDTA) through the coupling reaction (Plu-EDTA microgels). The created microgels had characteristics such as high affinity for curcumin (a type of hydrophobic drug), temperature- and pH dependent diffusion; As a result, curcumin was effectively loaded inside these microgels and its release in 37 °C was sustained and controlled. Additional investigations showed that these curcumin-containing microgels have cytotoxic effects on MDA-MB-231 breast cancer cells and can also release more curcumin in acidic environments that similar to the tumor microenvironment (Fig. 2D) [95].

In an innovative study conducted in 2016 by Su et al., pluronic-based micelles loaded with PTX were coated using red blood cell (RBC) membranes. Then, their characteristics and their effects on the mouse model for metastatic breast tumor were investigated. The results showed that this formulation (RBC-mimetic vesicle coated hybrid polymeric nanoparticles or RVPNs) compared to hybrid polymeric nanoparticles containing PTX without RBC membrane coating, prolongs the circulation time of nanoparticles in the blood and increases their biocompatibility. It was also found that simultaneous administration of RVPNs with iRGD (a type of peptide used to penetrate tumors) increases the penetration of these nanoparticles into primary tumors. In addition, it was found that RVPNs + iRGD have much higher anti-growth and anti-metastatic effects compared to the use of polymeric nanoparticles containing PTX or the combination of polymeric nanoparticles with iRGD peptide. In other words, we can say that covering polymeric nanoparticles with RBC membrane improves drug delivery into tumors composed of 4T1 breast cancer cells (Fig. 2E) [96].

### 4.2 Lung cancer

In this section, examples of the use of pluronics in researches related to lung cancer, which is one of the deadliest types of cancer [97], will be mentioned.

In the study conducted by Patel et al., it was observed that the use of Poly Caprolactone (PCL)/pluronic F68 nanoparticles can be effective in optimizing the use of Silibinin (SB). Silibinin is a chemically specified plant-based compound with anticancer properties; However, the low solubility of this drug in water as well as its low absorption rate after oral administration have limited its clinical use. In this study, it was found that PCL/pluronic F68 nanoparticles loaded with Silibinin have properties such as long blood circulation and successful tumor targeting. Also, PCL/pluronic F68 nanoparticles improved the bioavailability of silibinin. In addition, SB-PCL/pluronic F68 nanoparticles after inhalation were able to significantly prevent tumor growth in rats in which lung cancer was induced [98].

In another study, pluronic P105 was used in combination with Vitamin E-d-α-tocopheryl polyethylene glycol succinate (vitamin E-TPGS) to improve the physicochemical properties of oleanolic acid (OA). OA is a natural triterpenoid that has an important role in the treatment of various types of cancers, but its characteristics such as low solubility in water, low permeability and high efflux limit its clinical application. For this reason, micelles composed of vitamin E-TPGS + pluronic P105 were used to improve these properties. After loading with OA, these micelles were introduced into non-small cell lung cancer cell lines. It was observed that the uptake of OA in this case, in A549 cells, is higher than the case where free OA was applied. In addition, in mouse models with tumors induced by A549 cells, it was observed that treatment with micelles containing OA can reduce the tumor size and increase the expression of pro-apoptotic proteins, which shows the better efficiency of OA loaded on micelles compared to free OA. Further in vitro studies on A549 and PC-9 cells also indicated a better effect of micelles loaded with OA compared to free OA [99].

Tong et al. used pluronic-containing complexes for better delivery of cisplatin. It was observed that nanocomplexes consisting of pluronic F127 and heparin, when combined with cisplatin, can reduce the cytotoxicity of cisplatin (compared to the free state), and maintain the antiproliferative effect of cisplatin on NCI-H460 lung cancer cells [100].

In a study by Chen et al. micelles composed of pluronics P105 and F127 were used to deliver docetaxel (DTX) drug to lung cancer cells resistant to this drug. It was observed that these DTX-loaded micelles can produce higher antitumor effects in taxol-resistant A549 lung cancer cells, compared to control samples (free drug). It was also found that the use of these micelles can significantly reduce the value of half-maximal inhibitory concentration (IC50) of DTX and also significantly increase the duration of its presence in the blood circulation [101].

**Fig. 3:**
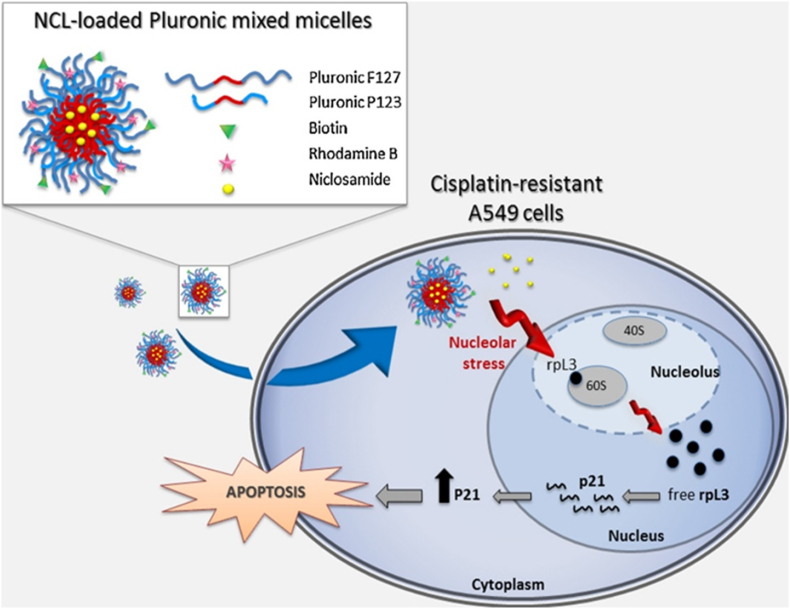
Structure of niclosamide-loaded pluronic micelles and their effects on cisplatin-resistant A549 cells. Adapted from Ref. [102] with permission, Copyright (2016), Elsevier.

In the study conducted by Russo et al., biotin-targeted pluronics loaded with niclosamide (NCL) were used as an alternative strategy to treat drug resistant lung cancer cells. For this purpose, two types of pluronic F127 and P123 were used; pluronic F127 was conjugated with biotin, while pluronic P123 was fluorescently tagged with rhodamine B (Fig. 3). Then, P123/F127 mixed micelles (PMM) were used to deliver niclosamide. In order to investigate the effects of NCL loaded Biotin-decorated PMM, A549 lung cancer cells that were resistant to cisplatin were used. First, these cells were treated with free NCL and it was observed that NCL has the ability to overcome the resistance to chemotherapy in these cells and causes cytotoxic effects; These effects included the increase of the ribosomal protein rpL3 and consequent upregulation of *p21*, which were related to the creation of nucleolar stress. Then, it was observed that NCL-loaded PMM nanocomplexes significantly can cause these toxic effects in lower doses in cisplatin-resistant A549 cells [102].

### 4.3 Cancers of gastrointestinal system

**Fig. 4:**
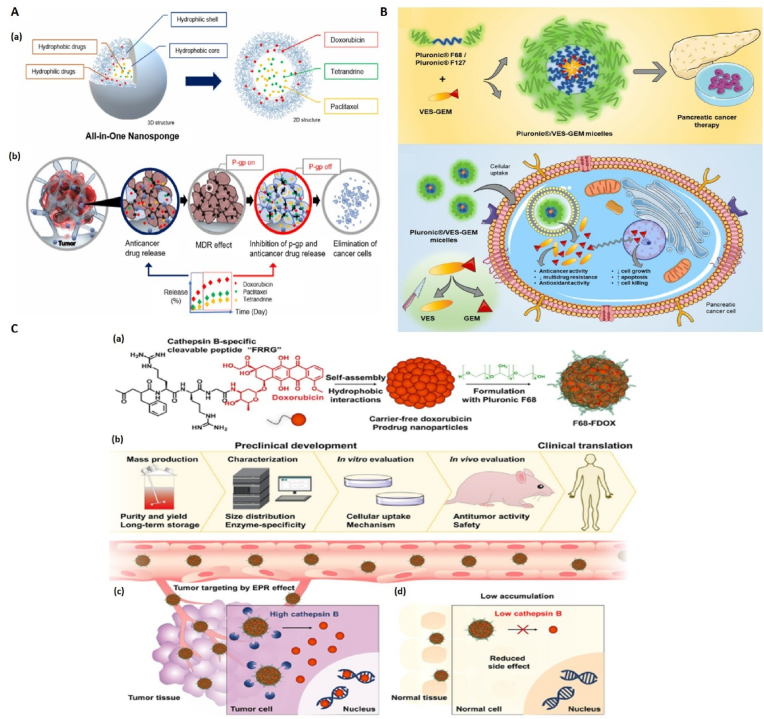
**Efficacy of ploronic-based nanocarriers for treatment of GI cancers**. **A,** (a). Structure of Pluronic-coated all-in-one **nanosponges for MDR colorectal cancer cells. (b). Effect of ANS on the inhibition of P-gp and the release of anticancer drugs**. Adapted from Ref. [109] with permission, Copyright (2022), Elsevier. **B,** Production mechanism of Gemcitabine-Vitamin E prodrug-loaded micelles (Pluronic®/VES-GEM) for pancreatic cancer therapy. Adapted from Ref. [115], Copyright (2024), MDPI. **C,** (a) Stimuli-responsive FRRG-DOX nanoparticles and their stabilization using Pluronic F68. (b) Hierarchy necessary to use this method in clinical applications. (c) Dissociation of DOX from FRRG and Pluronic units as a result of the presence of high amounts of cathepsin B in tumor cells. (d) DOX is not dissociated from FRRG and Pluronic units due to the presence of low levels of cathepsin B enzyme in normal cells. Adapted from Ref. [117] open access, Copyright (2022), Springer Nature.

Gastrointestinal (GI) cancers actually refer to malignancies in the oral cavity, colorectal, stomach, pancreas, gallbladder, liver, and esophagus. Cancers of the GI system are among the most common cancers and cause a high number of deaths worldwide [103,104]. In this section, the use of pluronics in researches related to cancers of the GI system is discussed (Fig. 4).

In the study conducted by Zhu et al., pluronic-based micelles were used to deliver 5-fluorouracil (5-Fu) to colon cancer cells. It was observed that when HCT116 and RKO colon cancer cells were treated with 5-Fu loaded on pluronic P85, 5-Fu could produce strong pesticide effects at lower doses, compared to the case without pluronic P85. Further investigations showed that the application of 5-Fu/P85 nanoparticles can reduce the migration and invasion ability of HCT116 cells and RKO cells. The general conclusion of this study was that Fu/P85 copolymer micelles can prevent the growth and metastasis of colon cancer [105].

Xie et al. used F127 pluronics along with electrospun fibrous meshes to deliver camptothecin (CPT) and curcumin (CUR) to colon cancer cells. For this purpose, different types of complexes including PF127-CPT-meshes, PF127-CUR-meshes and PF127-CPT/CUR-meshes, and with different weight ratios were made. The distribution of drugs inside the meshes and their release from the meshes were desirable. The results showed that these complexes have antitumor effects against colon cancer in vitro and it was also found that when using PF127-CPT/CUR-meshes, these antitumor effects are also synergistic [106].

Chitosan-based nanoparticles and pluronics can increase the loading amount of metformin hydrochloride (MF); This was one of the findings from the study by Arafa and colleagues. MF is proposed as an adjuvant drug for the treatment of colorectal cancers, but this drug is trapped in nanoparticles in a small amount. In order to solve the mentioned problem, in this study, a semi-interpenetrating network (semi-IPN) was created using chitosan and pluronic-123 micelles. The results showed that the use of this system can increase the rate of entrapment and continuous release of MF, thus increasing the sensitivity to treatment in RKO colorectal cancer cells [107].

In the study conducted by Correia, micelles based on pluronics were used for the co-delivery of SN-38 and lonidamine (LND) drugs to the lovo cell line (related to colon adenocarcinoma). These micelles were a combination of P123 and F127 pluronics containing different proportions of SN-38 and LND drugs. The created micelles had favorable physical and chemical properties and the corresponding CMC value was 0.019 mg/mL. Also, the continuous release of LND through these micelles was better compared to free drug (55 % and 97 % in 30h, respectively) [108].

Lee et al. used pluronic-coated all-in-one nanosponges (ANS) for drug delivery to MDR colorectal cancer cells. These integrated nanosponges contained tetrandrine (TET), doxorubicin and paclitaxel (DOX/PTX/TET@ANS) [109]. TET was used to inhibit P-glycoprotein (or P-gp is a type of transporter that plays an important role in resistance to most chemotherapy drugs [110]). ANSs had favorable characteristics such as high drug loading capacity and pH-dependent and controlled release. The results of in vivo studies on mice carrying colorectal tumor cells with overexpression of P-gp showed that DOX/PTX/TET@ANS formulations have higher anticancer effects compared to the mentioned drugs in free form as well as DOX/PTX@ANS formulation. Also, the results of in vitro studies indicated that ANS with pluronic shell has a higher inhibitory effect on P-gp compared to free TET (Fig. 4A) [109].

Pluronic-based nanoparticles were used by Yang et al. to deliver sorafenib to BGC-823 gastric cancer cells. These nanoparticles consisted of pluronic F-68 (Mw = 8350; (EO)79(PO)28(EO)79)) coated with salicylic acid-chitosan (SAC) and heparin salt and were loaded with sorafenib. Nanoparticles had favorable physicochemical properties. Further investigations showed that heparin-functionilized nanoparticles containing sorafenib have higher anticancer effects and higher apoptosis rates in BGC-823 cells; Compared with the situation using free sorafenib. The mentioned effects were also observed in xenograft tumor models [111].

Cubosomes, in drug delivery, are defined as biocompatible carriers that are nanostructured liquid crystal particles that are composed of specific amphiphilic lipids in different proportions [112]. In this line, Kumar et al., suggested that cubosome system based on pluronoics can be effective in the treatment of gastric cancer. They found that the use of cubosomes composed of pluronic F-127 and pluronic F68 for DTX drug delivery can improve the continuous and stable release of the drug as well as more accurate delivery of DTX [113].

In the study conducted by Chauhan et al., it was found that the use of nanoformulations consisting of pluronics and the drug Ormeloxifene (a non-steroidal, non-hormonal selective estrogen receptor modulator (SERM)) can be effective in the treatment of pancreatic cancers. In this research, different types of pluronics were used for formulation; but the most optimal ones were related to pluronics F127 and F68. As a result of this study, it was found that these nanoformulations containing Ormeloxifene are more uptaked by cells and have more anticancer effects on MiaPaCa and HPAF-II pancreatic cancer cell lines, compared to free Ormeloxifene [114].

In the study of Silva et al., in order to increase the effectiveness of chemotherapy using Gemcitabine (GEM) and to optimize its performance, pluronic-based micelles were used. GEM has a hydrophilic structure, which makes its encapsulation inside micellar nanocarriers difficult. On the other hand, the use of this drug in free form is associated with limitations such as short half-life, inability to target and systemic toxicity inside the body. In order to overcome the aforementioned limitations, GEM was first conjugated with vitamin E succinate (VES) to make VES-GEM conjugate and obtain hydrophobic properties. Then this compound was encapsulated inside micelles based on pluronics F68 and F127 separately (pluronic F68/VES-GEM and pluronic F127/VES-GEM) (Fig. 4B). VES-GEM loaded micelles showed an encapsulation efficiency of more than 95 % and had a favorable size. In vivo studies on pancreatic cancer cell line BxPC3 showed that both pluronic F68/VES-GEM and pluronic F127/VES-GEM micelles have higher cytotoxicity on cancer cells compared to blank micelles and GEM in free form. In addition, it was found that the stability of pluronic F127/VES-GEM micelles is higher compared to pluronic F68/VES-GEM micelles [115].

In a new research, Mdlovu and colleagues designed and manufactured nanocarriers known as MSNs@P123, in order to deliver drugs to liver cancer cells. Pluronic P123 was used to make these nanocarriers. In fact, pluronic P123 was used along with MCM-41 mesoporous material to cover the core consisting of magnetic iron oxide nanoparticles (MIONs). These nanocarriers are actually considered as a stimuli-responsive system. Then the nanocarriers were loaded with DOX. The use of this system improved the rate of DOX loading and temperature -dependent release, as well as more targeted drug delivery. Also, additional in vitro on liver cancer cells and in vivo studies showed that these DOX-loaded systems have high anticancer activity and effects [116].

In the study conducted by Shim et al., pluronic-based formulations were used to deliver the prodrug DOX to cancer cells. The said prodrug was formed by connecting DOX to a peptide that can be broken by cathepsin B enzyme (consisting of amino acids Phe-Arg-Arg-Gly; FRRG) and then was stabilized by F68 pluronics. DOX-FRRG combination has self-assembly feature (Fig. 4C). The effects of the resulting nanoparticles were investigated in vitro and in vivo on colon (HT29), pancreatic (KPC960) and breast (MDA-MB-231) cancer cells. In these investigations, the principle that the amount of cathepsin B enzyme is higher in cancer cells than in normal cells was used, as a result of cathepsin B enzyme activity in tumor cells, the peptide tail is separated and DOX is released. The results of this study indicated that the F68-FDOX formulation efficiently accumulates inside the tumor tissue and at the same time shows a very low toxicity in normal mice (compared to free DOX). In addition, in this research, a carrier-free approach was used to transfer the DOX prodrug to cancer cells, which is an alternative preclinical method and potentially has the ability to be generalized to the clinic (Fig. 4C) [117].

### 4.4 Prostate cancer

Prostate cancer is the most common cancer diagnosed in men and accounts for 15 percent of all cancers [118,119]. In this section, a number of studies on the use of pluronics in the treatment of prostate cancer are discussed.

In a study conducted by Nasehi et al., micelles consisting of pluronic F127 along with lithocholic acid were used to deliver sorafenib to prostate cancer cells. Application of micelles loaded with sorafenib to RBCs indicated that these micelles have high hemocompatibility properties. Also, micelles without drugs did not cause any toxicity in control cells. However, when LNCaP and DU145 prostate cancer cell lines were treated with sorafenib-loaded micelles, the antiproliferative effects observed were greater than when sorafenib was applied alone [120].

Cabazitaxel (CTX) is a very effective drug for the treatment of prostate cancer, but some of its physical and chemical characteristics, such as low solubility and imprecise targeting, limit the use of this drug. In order to overcome this problem, Jangid et al. conducted a study and created a CTX prodrug using pluronic F68 and pH-sensitive linkers (cis-aconityl (CAA) and succinoyl (SA) linkers). These prodrug nanomicelles had favorable physicochemical properties, such as low CMC. Two different formulations of nanomicelles were F68-SA-CTX and F68-CAA-CTX. It was observed that F68–CAA–CTX nanomicelles respond better to pH and exhibit more controlled drug release compared to F68–SA–CTX. Also, F68-CAA-CTX had a great ability to inhibit growth and induce apoptosis in prostate cancer cells. In general, the results of this study indicated that the application of CTX as prodrugs consisting of pluronic nanomicelles has a better effect compared to the conventional application of this drug [121].

**Fig. 5:**
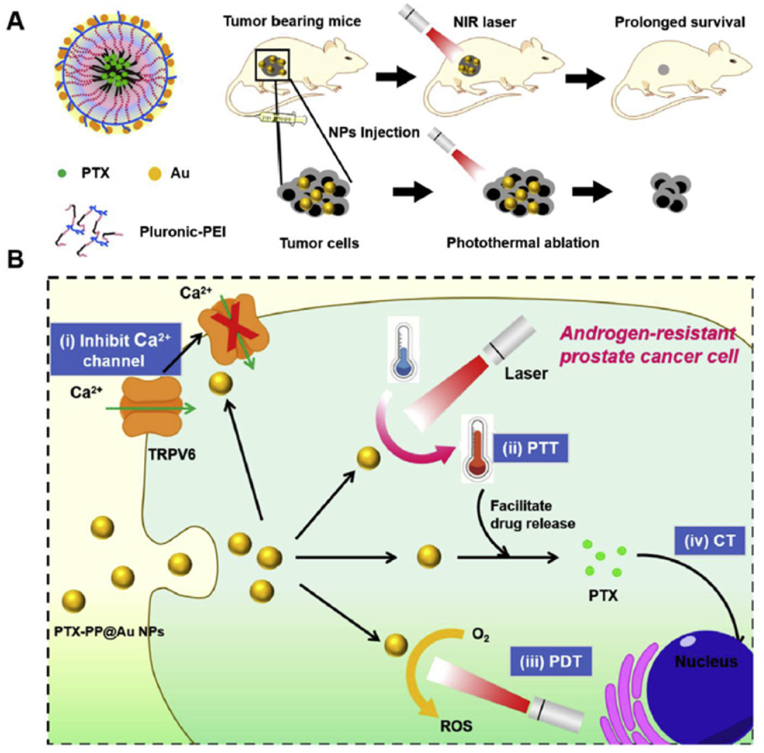
**Ploronic nanocarriers for treatment of Prostate cancer. A,** The structure of PTX-PP@Au nanoparticles, their transfer to a mouse model and the implementation of photothermal therapy using Near-infrared (NIR) laser. **B,** Multiple functions of PTX-PP@Au nanoparticles in ARPC cells. Adapted from Ref. [122] with permission, Copyright (2019), Elsevier.

In order to target androgen-resistant prostate cancers (ARPC), Wang et al. designed a multifaceted platform based on nanoparticles. These nanoparticles consisted of pluronic F127, polyethylenimine (PEI) and gold particles. First, pluronic-PEI (PP) nanoparticles were formed and loaded with PTX; Then gold particles were added to them. The resulting nanoparticles (PTX-PP@Au) were introduced into ARPC cancer cells. This platform was used to simultaneously perform PTT, PDT, effective transfer of PTX and blocking of TRPV6 calcium ion channel (Fig. 5). In vitro studies showed that nanoparticles PTX-PP@Au had the ability to block the TRPV6 ion channel and increase cell cycle arrest, and their cellular uptake was also high; These factors lead to an increase in cytotoxicity and apoptosis. In general, the results of this research indicated that the use of this multifaceted platform can inhibit the growth of ARPC cancer cells through the synergy caused by the use of 4 different treatment strategies against cancer cells, and at the same time cause the least side effects [122].

### 4.5 Ovarian and uterine cancers

**Fig. 6:**
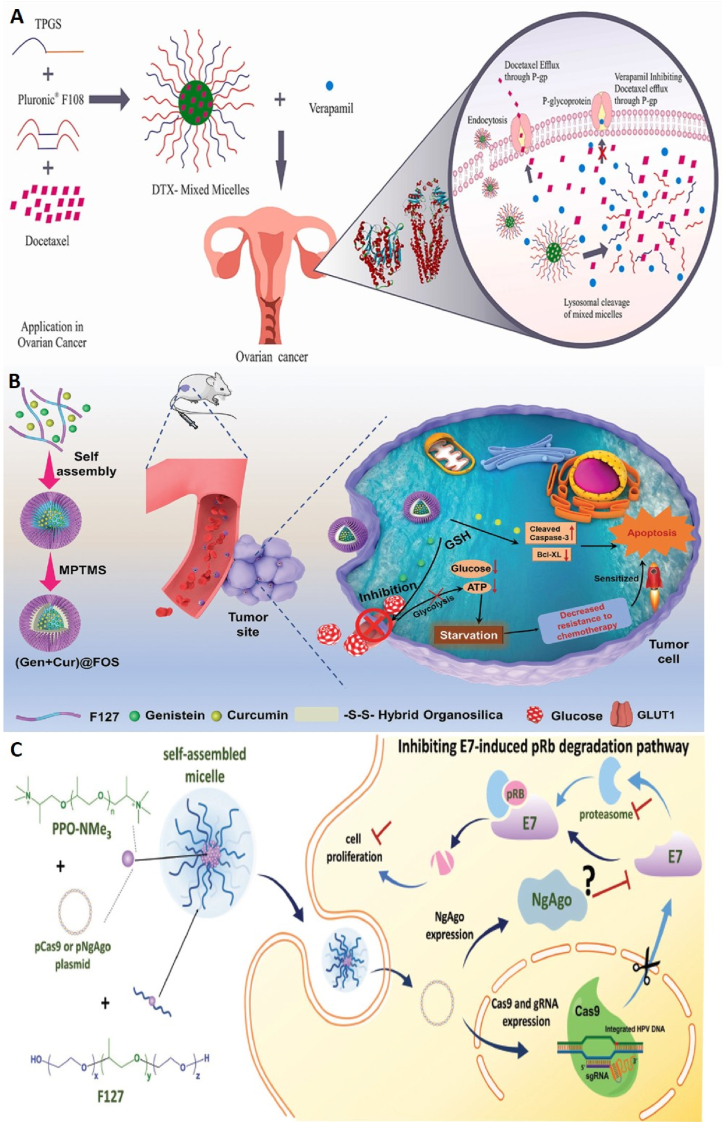
**Ploronic nanocarrier for treatment of female reproductive system cancers. A,** Schematic of DTX-Mixed Micelles formulation. Transfer of DTX-Mixed micelles nanoparticles with verapamil to ovarian cancer cells and their synergistic effects. Adapted from Ref. [126] with permission, Copyright (2021(, Elsevier **B,** Schematic of the structure of (Gen + Cur)@FOS nanoparticles and their "valve-closing" effect on Hela cells. Gen increases the sensitivity of tumor cells to Cur through GLUT1 inhibition. Adapted from Ref. [127] Copyright (2022), John Wiley and Son. **C,** Delivery of pCas9 or pNgAgo plasmids loaded inside F127/PPO-NMe_3_ micelles into HeLa cells and their inhibitory effect on the cell proliferation pathway induced by HPV18-E7 oncogene in these cells. Adapted from Ref. [128] Copyright (2018), John Wiley and Son.

Ovarian and uterine cancers are the eighth most common cancer in women and have a global prevalence. In 2020, 4.7 % of cancer deaths are related to this type of cancer [123,124]. In the following, examples of studies conducted regarding the use of pluronics in the field of ovarian and uterine cancers are given (Fig. 6).

Luo et al. synthesized folate (FA)-pluronic F127-PLGA and PLGA-F127-PLGA block copolymers and used the nanoparticles obtained from each of these copolymers to encapsulate PTX. Both nanoparticles were prepared by dialysis method and PTX was successfully loaded inside them. In vitro cytotoxicity studies on OVCAR-3 cells showed that when PTX was encapsulated inside FA-F127-PLGA and PLGA-F127-PLGA nanoparticles, its antitumor effects increased compared to free PTX. Since OVCAR-3 cells overexpress the folate receptor, it was found that the use of FA-F127-PLGA nanoparticles was more effective than PLGA-F127-PLGA nanoparticles for the delivery of PTX (due to receptor-mediated endocytosis). As treatment time increased, this difference was more obvious. In addition, the in vitro investigations confirmed that FA-F127-PLGA nanoparticles more easily uptaked by OVCAR-3 cells compared to PLGA-F127-PLGA nanoparticles. Pharmacokinetic studies also revealed that FA-F127-PLGA nanoparticles delayed the blood clearance of PTX and increased its circulation time in plasma [125].

DTX is one of the chemotherapy drugs for various types of cancers; however, due to many side effects, multi-drug resistance and poor solubility, its application faces limitations. To solve these limitations, Patil and colleagues formulated DTX inside pluronic-based micelles. These mixed micelles (MMs) consisted of pluronic polymer F108 and tocophersolan (TPGS) loaded with DTX (DTP MMs). For this purpose, the interaction of DTX with the hydrophobic core of MMs was investigated in silico; after confirming the proper interaction of DTX with the core of micelles, these nanoparticles were synthesized and after analyzing the physicochemical properties, were used for in vitro studies. Compared to free DTX, DTP MMs showed characteristics such as sustained release, reduced destruction of blood cells and less in vitro cytotoxicity. Then, the effects of DTP MMs alone and together with Verapamil (VPM) on SKOV-3 ovarian cancer cell line were investigated (Fig. 6A). The results showed that compared to free DTX and DTP MMs, the addition of VPM at low concentrations increases the inhibitory effect on SKOV-3 cells, while no such effect was observed at high concentrations of VPM [126].

Li et al. used a novel starvation strategy called “valve closing” to increase the sensitivity of tumor cells to chemotherapy and reduce its side effects. In this study, an inhibitor of glucose transporter 1 (GLUT1), called genistein (Gen), was used to block the glucose transport “valve” in Hela cells. Gen and curcumin were co-loaded inside pluronic F127 micelles. In addition, (3-mercaptopropyl)trimethoxysilane (MPTMS) was subsequently used to endow the micelles with controllable stability (i.e., circulating biostability and stimuli-responsive biodegradability). Hydrolysis/condensation of MPTMS in micellar cores creates an inorganic-organic hybrid network containing disulfide bonds. Therefore, hybrid organosilica-micelles nanocarriers (FOS) loaded with Gen and Cur ((Gen + Cur)@FOS) were obtained. In vitro and in vivo studies on HeLa cells and mice with tumors derived from HeLa cells showed that (Gen + Cur)@FOS can significantly reduce the level of glucose and adenosine triphosphate in tumor cells through the inhibition of GLUT1 expression (“valve-closing”). This leads to starvation in tumor cells and weakens the resistance of these cells against apoptosis caused by chemotherapy (Fig. 6B). Therefore, due to the sensitization effect induced by starvation stress, the antitumor efficiency of chemotherapy increases and its side effects decrease [127].

CRISPR/Cas9 is a technology for targeted gene editing and has found many applications in cancer studies. However, plasmid-based CRISPR/Cas9 delivery with non-viral carriers is associated with low efficiency and specificity. To overcome this limitation, Lao et al. designed and synthesized a novel non-viral delivery system to deliver plasmid-based gene editing and manipulation systems to cancer cells. This system was a self-assembled micelle composed of quaternary ammonium-terminated poly(propylene oxide) (PPO-NMe_3_) and pluronic F127 (F127/PPO-NMe_3_). In this study, micelles were optimized to deliver an all-in-one Cas9 construct (called pCas9) into HeLa cells. pCas9 encodes both Cas9-green fluorescent protein (GFP) and guide RNA (gRNA) against HPV18-E7 (a human papillomavirus oncogene). In addition, the potential of F127/PPO-NMe_3_ micelles to deliver another nucleic acid-guided nuclease system, *Natronobacterium gregoryi* Argonaute (NgAgo) enzyme to the same cancer model was investigated. The results showed that the micelles containing pCas9 effectively knocked out the E7 oncogene in Hela cells and thus significantly inhibited the cancer activity caused by HPV both in vitro and in vivo. However, it was found that NgAgo does not have significant inhibition on E7 oncogene in xenograft mouse model (Fig. 6C) [128].

### 4.6 Skin cancers

**Fig. 7:**
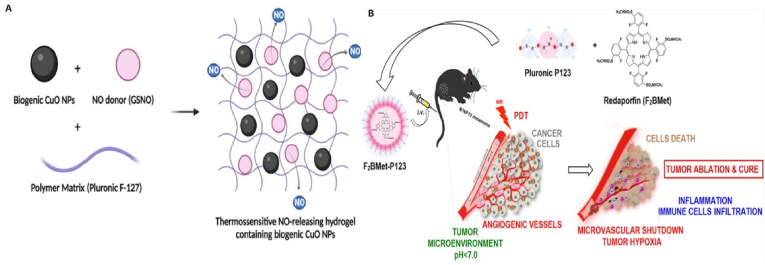
**Ploronic nanoparticles for treatment of skin tumors. A,** Schematic structure of Pluronic F-127 hydrogel containing GSNO and CuO NPs for local therapy of B16F10 melanoma cancer. Adapted from Ref. [130] Copyright (2023), MDPI. **B,** Schematic representation of F_2_BMet-P123 micelles preparation and their effect on increasing PDT efficiency on B16F10 melanoma tumor cells. Adapted from Ref. [132] with permission, Copyright (2016), American Chemical Society.

Skin cancer is actually the most common type of cancer in the world; The prevalence of this type of cancer has also increased during the last decade [129]. Various studies have been conducted regarding the use of pluronics in skin cancer research (Fig. 7), some of which are mentioned below.

The use of pluronic in studies related to skin cancers has mainly been in the form of hydrogels/nanogels carrying therapeutic compounds. Due to their high biocompatibility properties, these polymeric hydrogels are suitable candidates for improving the application of local therapy and can reduce limitations such as skin irritation and side effects caused by non-invasive topical treatments. Cabral et al. synthesized pluronic F-127 (PL) hydrogels containing nitric oxide donor S-nitrosoglutathione (GSNO) and copper oxide nanoparticles (CuO NPs) (Fig. 7A) and investigated its therapeutic effects on murine B16F10 melanoma cells. The results show that in comparison with other groups (PL, PL + GSNO, PL + CuO NPs), PL + GSNO + CuO NPs significantly reduce the viability of B16F10 cells and increase the level of reactive oxygen species to a great extent. It was also found that PL + GSNO + CuO NPs formulation can lead to nuclear changes, disruption of mitochondrial membrane potential and lipid peroxidation through the effect on cell organelles which induces cell death [130].

Kim et al. synthesized thermosensitive hydrogels composed of gelatin and pluronic F127 (F127-g-gelatin polymer) and used it as a co-delivery system to perform immunochemotherapy for the treatment of BRAF-mutated melanoma tumors. First, these hydrogels were loaded with antagonistic programmed cell death protein 1 antibody (aPD-1) as a checkpoint blockade immunotherapy agent and BRAF inhibitor vemurafenib as a chemotherapy agent. Then, the antitumor effect of the mentioned formulation was investigated in murine models of BRAF-mutated melanoma. The results of in vivo evaluations revealed the potent antitumor effects of this combination therapy. It was also found that F127-g-gelatin hydrogel facilitates the long-term local drug release within the tumor and improves local immunomodulation, tumor response rate, tumor inhibition and consequently animal survival. Therefore, compared to the treatment with each of free vemurafenib and free aPD-1, F127-g-gelatin hydrogel can improve the effectiveness of these compounds in the treatment of BRAF-mutated melanoma in a local chemoimmunotherapy approach [131].

Pucelik et al. used pluronic micelles to improve the efficacy of PDT with redaporfin (F_2_BMet) (Fig. 7B). Redaporfin molecules were loaded inside pluronic micelles (P123 and F127) and the inhibitory effects of these formulations on melanoma cells and tumors were investigated in the dark and during PDT. The results of in vitro cytotoxicity showed that in the dark state redaporfin encapsulated in pluronic micelles and the micelles alone do not have toxicity on B16F10 melanoma cells (especially in lower concentrations). However, during PDT, redaporfin, redaporfin-P123 and redaporfin-F127 formulations showed different cytotoxicities on these cells, and the highest level of cytotoxicity was related to redaporfin-P123. It was found that redaporfin-P123 micelles increased cell uptake by B16F10 cells and compared to redaporfin-F127 and redaporfin alone, it caused more efficient formation of hydroxyl radicals in cancer cells and enhanced oxidative stress. The results of in vivo studies on mice bearing B16F10 melanoma tumors revealed that pluronic P123 formulation of redaporfin increases its bioavailability in tumors compared to pluronic F127 and Cremophor EL formulations. Compared to other formulations, redaporfin-P123 was the most successful in inhibiting the growth of melanoma tumors, so that during Vascular-targeted PDT, the concentration of 1.5 mg kg^−1^ of redaporfin loaded in P123 with an optical dose of 74 J cm^−2^ led to complete cure [132].

### 4.7 Brain tumors

Brain cancer is one of the most common cancers worldwide and affects about 250,000 people every year [133]. One of the reasons that make the treatment of this tumors difficult is the presence of the BBB; Because it reduces the efficiency of drug delivery to the tumor tissue in the brain [134].

**Fig. 8:**
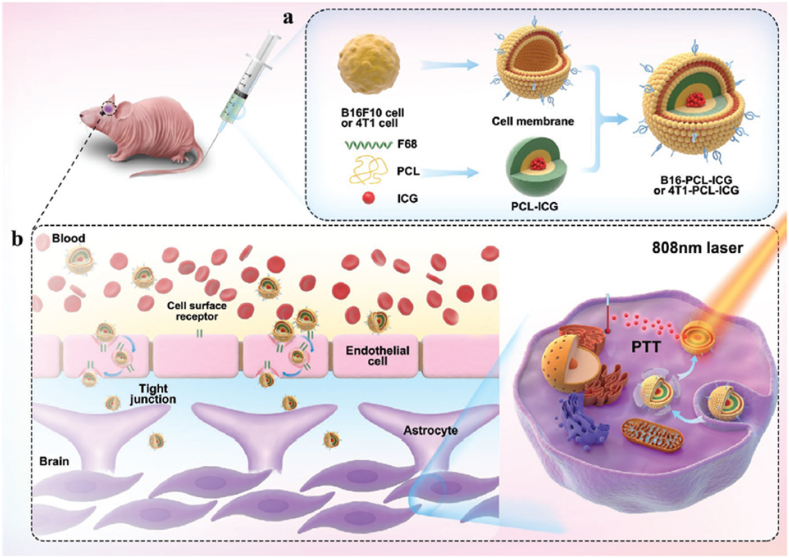
**Ploronic nanoparticle for treatment of brain tumors**. a. Structure of nanoparticles consisting of PCL-ICG core and membrane coating extracted from cancer cells. b. The ability of manufactured nanoparticles to cross the BBB and their application in photothermal therapy. Adapted from Ref. [135] with permission, Copyright (2020), John Wiley and Sons.

In the study conducted by Wang et al., nanoparticles composed of PCL and pluronic F68 containing indocyanine green (ICG) and covered with the membrane of cancer cells were used to overcome the BBB. In this study, PCL-ICG polymer nanoparticles were encapsulated using the cell membrane extracted from B16F10 mouse melanoma cancer cells and 4T1 breast cancer cells (separately) (Fig. 8). The goal was that these nanoparticles can cross the BBB and have the ability to be used for imaging and PTT. U87MG human glioblastoma cells were treated with different formulations of nanoparticles. The results showed that both the BBB penetration and the anti-growth ability of biomimetic B16-PCL-ICG and 4T1-PCL-ICG nanoparticles are higher compared to PCL-ICG formulations alone and PCL-ICG coated with normal cell membrane [135].

## 5 Pluronics application: advantages and challenges

Extensive research has been conducted to evaluate the safety profile, immunogenic properties, and metabolic clearance of pluronics. These studies have demonstrated that pluronics exhibit hemorheological benefits by reducing blood viscosity, inhibiting erythrocyte aggregation, and mitigating cellular adhesion to the vascular endothelium. Furthermore, evidence suggests that pluronics enhance microcirculatory hemodynamics, thereby improving perfusion within the microvascular networks of the body [17].

In addition, pluronic micelles, owing to their nanoscopic dimensions, are capable of evading recognition and clearance by the reticuloendothelial system (RES), thereby prolonging their systemic circulation. Furthermore, the plasma circulation of these nanoparticles can be enhanced through modifications applied to their surface properties. Another advantageous characteristic of these nanoparticles is their ability to facilitate the formulation of water-soluble drugs, poorly water-soluble drugs, and even a combination of both types [12].

Despite the remarkable advantages highlighted for the application of Pluronics, their use can occasionally be accompanied by some challenges. For example, repeated administration of pluronic-based nanoparticles has been shown in some studies to elicit an adaptive immune response, resulting in the generation of anti-polymer antibodies. These antibodies interact with and facilitate the clearance of subsequent nanoparticle doses, thereby attenuating their therapeutic efficacy [12]. Another challenge in the application of pluronics is the use of PEG-conjugated pluronics, which induces the production and accumulation of anti-PEG antibodies. This immune response can lead to the systemic clearance of these nanoparticles, as well as other nanoparticle-based therapeutics, thereby compromising their overall efficacy [136].

Furthermore, the heterogeneity in the physicochemical attributes of pluronic-based nanoparticles such as molecular weight, hydrophobic-hydrophilic balance, and surface characteristics can significantly influence their colloidal stability, drug release profiles, and target-specific delivery efficiency [12]. Moreover, due to the hydrophilic surface of pluronics, pluronic formulations exhibit limited tumor selectivity and penetration within tumor cells. Nevertheless, this can naturally be optimized by employing different functionalization strategies [137].

Another challenge associated with the use of pluronics is their sensitivity to environmental changes. Variations in pH levels and potential interactions with various molecules, such as proteins, lipids, and different types of cells, can disrupt the functionality and efficacy of Pluronic micelles [36]. Therefore, when utilizing pluronics, it is essential to consider this aspect and implement the necessary optimizations to address these challenges effectively.

## 6 Pluronics; from bench to bedside

Two pluronic copolymers named pluronic F-68 (poloxamer 188) and F-127 (poloxamer 407) have succeeded in obtaining FDA approval for pharmaceutical and biomedical applications in living organisms [12]. Today, several products based on these two copolymers are waiting for FDA approval to enter the market (information adapted from https://www.fda.gov/). Our searches in the relevant internet sources, showed no approved pluronic-based medicinal product for the specific treatment of cancer so far.

However, pluronics can be effective in the cancer treatment process from various other aspects; Some formulations based on these copolymers have been studied to be used alongside the main treatment for faster patient recovery and also to reduce the side effects that occur during or after cancer treatment. For example, in a clinical trial conducted in collaboration with Samsung Medical Center in 2017, the combination of poloxamer, gelatin and chitosan (under the name Mediclore®) was used to reduce adhesions after axillary lymph node surgery in people with breast cancer and restore shoulder movements (NCT02967146). The results of these studies showed that the aforementioned formulation can significantly reduce the movement restrictions that occur after lymph node surgery in the shoulders in the first four weeks compared to the control group [112]. However, these effects are not definitive, and for example, the effectiveness of Mediclore® in improving postoperative adhesions after thyroidectomy is debatable [138].

In another clinical trial conducted in 2021 (NCT04070677), ZIVEREL® (consisting of hyaluronic acid, chondroitin sulfate, and poloxamer 407) was used to relieve the symptoms of esophagitis developed after radiotherapy or chemoradiotherapy in cancer patients. Poloxamer 407 acts as a bioadhesive in this formulation. The results of this clinical trial have not yet been made public. However, in some cases, the effectiveness of ZIVEREL® formulation in improving the symptoms of some disease conditions, including reflux, is not significant, making the use of this drug questionable [139].

## 7 Artificial intelligence and pluronics

In recent years, artificial intelligence (AI) and its various subfields, such as machine learning (ML) and artificial neural networks (ANN), have found applications in diverse scientific disciplines. In the field of nanomaterials, the potential of artificial intelligence can also be harnessed in various aspects. For example, machine learning algorithms can be utilized to optimize the bioprinting parameters of various nanomaterials, including pluronics [140]. Furthermore, the potential of machine learning can be harnessed to predict the printability properties of nanomaterials, including pluronics [141]. Moreover, machine learning models, such as support vector machine (SVM) model, can be utilized to develop systems that reduce time and facilitate the formulation process of pluronic-based nanoparticles [142]. Additionally, machine learning models can be employed to investigate the distribution of polymer conformations within the micelles [143]. Optimizing the percentage of pluronic-based drug-loaded compositions is another example of the application of artificial intelligence systems, particularly artificial neural networks, in this field [144].

In summary, it can be envisioned that in the future, using the potential of artificial intelligence, the process of synthesis, optimization, and even the examination of the interactions of pluronics can be accelerated and improved.

## 8 Conclusion

Pluronics or poloxamers are synthetic and amphiphilic three-block copolymers that are composed of hydrophilic PEO and hydrophobic PPO blocks in the form of (PEO)_n_-(PPO)_m_-(PEO)_n_. Desirable physicochemical properties and features such as biocompatibility, non-toxicity on normal cells and tissues, as well as their therapeutic effects in some diseases, have caused special attention to these copolymers in biomedical research.

Among the various fields of biomedical studies, the most common application of these copolymers is in research related to regenerative medicine, tissue engineering, and cancer treatment. In this review, we discussed a number of cases of using pluronic formulations in researches related to breast, lung, digestive system, prostate, uterus, ovary, skin and brain cancers (Table 2). In this researches, pluronic-based complexes were generally used for more efficient and effective drug delivery to cancer cells.

**Table 2:** Various pluronic-based formulations and their research applications in different types of cancers.

Cancer type	Pluronic formulation	Loaded material
Breast	Gold nanoparticle-coated pluronic-b-poly(L-lysine) nanoparticles	PTX
	Pluronic CuTz-1-O2@F127	Cu(I) and O_2_
	Pluronic-lipid nanovesicle hybrids (LPL)	PTX
	Hyaluronic acid (HA)-conjugated P123 and F127 pluronics	Thymoquinone (TQ)
	Combination of L61 and F127 pluronics	DOX
	Phenylboric acid (PBA)-modified F127 and doxorubicin-grafted P123	DOX
	Pluronic P123 was modified with α-tocopherol succinate	DOX
	Pluronic F68 (PF68)-attached polyamidoamine (PAMAM) dendrimer conjugates	DOX
	Folate-conjugated chitosan-pluronic P123 nanogels	PTX and Curcumin
	Pluronic-chitosan-folate nanomicelles	Quantum dots (QD) and DOX
	Pluronic F127 (PF127) and pluronic F68 (PF68) micelles	Vorinostat
	Pluronic F127 (PF127)-folic acid (FA) conjugated micelles	Fisetin (FS)
	Plu-EDTA microgels	Curcumin
	F68 pluronic coated with RBC membrane	PTX
Lung	PCL/pluronic F68	Silibinin (SB)
	Pluronic P105 with Vitamin E-d-α-tocopheryl polyethylene glycol succinate	Oleanolic acid (OA)
	Pluronic F127 and heparin	Cisplatin
	Pluronics P105 and F127	DTX
	Biotin-targeted pluronics F127 and P123	Niclosamide (NCL)
Gastrointestinal	Pluronic P85	5-fluorouracil (5-Fu)
	F127 pluronics along with electrospun fibrous meshes	Camptothecin (CPT) and Curcumin
	Chitosan and pluronic-123 micelles	Metformin hydrochloride (MF)
	Combination of P123 and F127 pluronics	SN-38 and Lonidamine (LND)
	Pluronic-coated all-in-one nanosponges (ANS)	Tetrandrine (TET), DOX and PTX
	Pluronic F-68 coated with salicylic acid-chitosan	Sorafenib
	Cubosomes composed of pluronic F-127 and pluronic F68	DTX
	Pluronics F127 and F68	Ormeloxifene
	Pluronics F68 and F127	Gemcitabine (GEM)
	Pluronic P123 with MCM-41 mesoporous material	DOX
	F68 pluronics	DOX
Prostate	Pluronic F127 along with lithocholic acid	Sorafenib
	Pluronic F68 and pH-sensitive linkers	CTX
	Pluronic F127 with polyethylenimine (PEI) and gold particles	PTX
Ovarian and Uterine	Folate (FA)-pluronic F127-PLGA and PLGA-F127-PLGA block copolymers	PTX
	Pluronic polymer F108 and tocophersolan (TPGS)	DTX
	Pluronic F127 micelles	Genistein (Gen) and Curcumin
	Pluronic F127	All-in-one Cas9 construct
Skin	Pluronic F-127 (PL) hydrogels	Nitric oxide donor S-nitrosoglutathione (GSNO) and Copper oxide (CuO)
	Gelatin and pluronic F127	Antagonistic programmed cell death protein 1 ntibody (aPD-1) and Vemurafenib
	P123 and F127 pluronics	Redaporfin
Brain	PCL and pluronic F68 covered with the membrane of cancer cells	Indocyanine green (ICG)

∗PTX: Paclitaxel, DOX: Doxorubicin, DTX: Docetaxel, CTX: Cabazitaxel, PCL: Polycaprolactone. Data obtained from Refs. [[82], [83], [84], [85], [86], [87], [88], [89], [90], [91], [92], [93], [94], [95], [96], [97], [98], [99], [100], [101], [102], [103], [104], [105], [106], [107], [108], [109], [110], [111], [112], [113], [114], [115], [116], [117], [118], [119], [120], [121], [122], [123], [124], [125], [126], [127], [128], [129], [130], [131], [132], [133], [134], [135]].

Compared to other drug delivery methods, one notable challenge associated with dendrimers is their toxicity, whereas this issue is not observed with pluronics, which exhibit very low toxicity [145,146]. In comparison to liposomes, a limitation of liposomes is their lack of on-demand content release) describes systems that deliver medication at the exact time and location required) [147,148], a property that pluronics possess. Regarding the stability of nanocarriers, while both liposomes and dendrimers display adequate stability, pluronics offer an advantage due to the greater possibility of surface modification, enabling tailored stability levels as desired. Furthermore, they possess the ability to encapsulate pharmaceutical agents for extended periods, ensuring sustained retention until they effectively reach their intended target site. Solid lipid nanoparticles (SLNs) and nanostructured lipid carriers (NLCs) are also among the other nanotechnology-based drug delivery methods [149]. However, these carriers face challenges such as limited drug-loading capacity and the potential of drug leakage. Conversely, such limitations are significantly less pronounced in pluronics. Additionally, a distinct feature of pluronics lies in their ability to self-assemble unimer molecules into micelles, a phenomenon referred to as micellization. This characteristic is not observed in dendrimers and liposomes [[150], [151], [152]].

Additionally, potential challenges concerning the safety, functionality, and efficiency of pluronics during their application were highlighted. The potential of pluronics for clinical applications, as well as the status of pluronics-related products in clinical trial research and their FDA approval status, was also emphasized. Considering the FDA approval of some of these copolymers for human use, as well as a wide range of researches, and the high potential of these molecules in improving drug delivery to cancer cells, it is hoped that in the near future a large number of formulations based on pluronics will succeed in obtaining relevant approvals for use in cancer treatment.

## CRediT authorship contribution statement

**Mehdi Pourbakhsh:** Writing – original draft, Visualization, Resources, Methodology, Investigation, Conceptualization. **Masoud Jabraili:** Resources, Methodology, Investigation. **Morteza Akbari:** Resources, Methodology, Investigation. **Mehdi Jaymand:** Validation, Software, Methodology, Investigation. **Rana Jahanban Esfahlan:** Writing – review & editing, Validation, Supervision, Funding acquisition, Conceptualization.

## Availability of data and materials

Not applicable.

## Funding

This study is supported by 10.13039/501100004366Tabriz University of Medical Sciences, grant number 71736.

## Declaration of competing interest

None to declare.

## Data Availability

No data was used for the research described in the article.
